# Assessing the real-world safety of vutrisiran for transthyretin-mediated amyloidosis with polyneuropathy: Based on WHO-VigiAccess and FAERS databases

**DOI:** 10.1371/journal.pone.0347417

**Published:** 2026-04-15

**Authors:** Weiwei Tong, Yaxing Li, Xingli Xu, Yang Mao, Ruonan Zhang, Chuanliang Zhao, Jidang Zhang, Chigang Du, Jiayue Feng

**Affiliations:** 1 Department of Clinical Laboratory, The Affiliated Taian City Central Hospital of Qingdao University, Taian, China; 2 Department of Cardiology, State Key Laboratory for Innovation and Transformation of Luobing Theory, Key Laboratory of Cardiovascular Remodeling and Function Research of MOE, NHC, CAMS and Shandong Province, Qilu Hospital of Shandong University, Jinan, China; 3 Department of Cardiology, Sichuan Provincial People’s Hospital, University of Electronic Science and Technology of China, Chengdu, China; 4 Department of Critical Care Medicine, Qilu Hospital of Shandong University, Jinan, Shandong, China; 5 Second Clinical Medical College, Shandong University of Traditional Chinese Medicine, Jinan, Shandong, China; 6 Department of Orthopedics, The Affiliated Taian City Central Hospital of Qingdao University, Tai’an, Shandong, China; 7 Medical Integration and Practice Center, Shandong University School of Medicine, Shandong University, Jinan, Shandong, P.R. China; 8 Medical School, Shandong University of Traditional Chinese Medicine, Jinan, China; 9 Department of Neurosurgery, The First Affiliated Hospital of Shandong First Medical University & Shandong Provincial Qianfoshan Hospital, Jinan, Shandong, P. R. China; Johns Hopkins: Johns Hopkins University, UNITED STATES OF AMERICA

## Abstract

**Background:**

Vutrisiran is a double-stranded siRNA targeting transthyretin mRNA, primarily for the treatment of transthyretin-mediated amyloidosis (ATTR) with polyneuropathy. Understanding its safety in real-world settings is crucial for achieving optimal treatment outcomes for patients.

**Methods:**

This study assessed the safety of vutrisiran for clinical use by analyzing all adverse drug reaction (ADR) reports in WHO-VigiAccess and U.S. Food and Drug Administration’s Adverse Event Reporting System (FAERS) databases with vutrisiran as the primary suspected drug and by using four disproportionality analysis and Bayesian information component. Weibull distribution was also utilized to predict the time of occurrence of ADR.

**Results:**

During the research process, WHO-VigiAccess reported 721 adverse reaction reports related to vutrisiran, while FAERS documented 732 ADR reports. The analysis revealed that: this study not only demonstrated known adverse reactions such as injection site reactions, such as redness, pain, or swelling at the injection site; systemic reactions like fatigue and myalgia; pain in extremity; and potential risks associated with reduced vitamin A levels, but also detected potential safety signals related to drug such as night blindness and cardiac pacemaker insertion. Furthermore, it was observed that in both the WHO-VigiAccess and FAERS databases, the highest reporting rates of ADR were found in the category of general disorders and administration site conditions. It also confirms the importance of monitoring the occurrence of adverse reactions within one year after drug treatment.

**Conclusion:**

The study identified several known ADRs and detected potential positive signals that may be associated with the drug. These research results provide clinicians with more information to consider and monitor adverse reactions in patients with ATTR-related polyneuropathy after treatment with vutrisiran, offering more evidence for its safety in real-world settings.

## Introduction

Vutrisiran is a double-stranded siRNA that targets both wild-type and mutant transthyretin (TTR) mRNA, [1] and is an FDA-approved drug for the treatment of hereditary ATTR-related polyneuropathy. TTR amyloidosis can be divided into two subtypes: non-hereditary wild-type ATTR and hereditary mutant ATTR. [2] Mutations in TTR can lead to misfolding and production of TTR protein, and these abnormal forms of the protein can form amyloid fibrils, which deposit and accumulate in various types of tissues in the human body (such as the heart and nervous system), causing damage to these tissues and posing a threat to human health. [3–5] Currently, the incidence and prevalence of transthyretin amyloid cardiomyopathy (ATTR-CM) and ATTR-related polyneuropathy in the population have been continuously increasing in recent years, leading to a decline in the quality of life for patients. [2] The molecular mechanism of vutrisiran targeting mRNA provides a new approach and perspective for the treatment of this disease.

TTR protein is mainly expressed in the liver, composed of 127 amino acids and exists in a tetrameric form. [6] Its main function is to transport vitamin A. More than 130 mutations of the TTR gene have been discovered. [7] The most common of these gene mutations is the substitution of valine with methionine at position 30 of the gene (Val30Met). [4] Once the TTR gene mutates, the tetrameric TTR protein dissociates into monomers, which then misfold into amyloid fibrils that deposit in other tissues such as the peripheral nervous system and heart. [3,4] Vutrisiran is a double-stranded siRNA (conjugated with N-acetylgalactosamine, which can target the corresponding receptors on liver cells) that targets and binds to the mRNA of both wild-type and mutant TTR, promoting its degradation, leading to a reduction in serum TTR protein levels and the amount of amyloid fibril deposition in ATTR patients, thereby exerting a therapeutic effect. [1,8] It has been approved by the FDA for the treatment of ATTR-related polyneuropathy. Currently, clinical trials for this drug in treating ATTR-CM have made significant progress and confirmed the drug’s efficacy. The HELIOS-B trial found that compared to the placebo group, ATTR-CM patients treated with vutrisiran had a lower risk of all-cause mortality and cardiovascular events, while maintaining quality of life and functional ability [9].

Although numerous clinical studies have demonstrated the efficacy of vutrisiran in patients, we must not overlook the adverse reactions associated with its long-term use. These adverse reactions not only pose unpredictable risks to patients’ health but may also impact their quality of life and impose additional economic burdens. Additionally, it is important to recognize that while clinical research is an indispensable tool for identifying drug-related adverse reactions, it cannot capture all real-world scenarios, such as the occurrence of rare ADRs. This is precisely the aim of the current study: to evaluate the safety profile of the drug by analyzing relevant data from databases and integrating findings from clinical studies, thereby addressing the aforementioned issues.

Spontaneous reporting systems (SRS) serve as a critical source for obtaining data on drug safety. The data analyzed in this study come from the WHO-VigiAccess and FAERS databases included in the SRS. The FAERS supports the FDA in monitoring safety reports for all marketed drugs and therapeutic biological products. It contains all ADR reports provided by doctors, consumers, and pharmacists. Despite certain inherent limitations, it remains an important resource for evaluating drug safety. [10] Numerous studies have utilized data from SRS databases to evaluate the real-world safety of various medications. For example, the FAERS database has been employed to investigate potential ADR associated with immune checkpoint inhibitors (ICIs) and selective serotonin reuptake inhibitors (SSRIs). [11,12] In addition, there are studies merging data from the WHO-VigiAccess and FAERS databases to explore the potential safety of three types of drugs: gepant drugs, antifibrotic drugs, and isocitrate dehydrogenase inhibitor drugs. [13–15]

This study conducts a disproportionality analysis using relevant data from the WHO-VigiAccess and FAERS database. It further evaluates the safety of vutrisiran in real-world settings and provides more safety evidence and clinical medication safety guidance.

## Materials and methods

### Data sources

The data used in this study were sourced from two databases: VigiAccess and FAERS. For the VigiAccess database, users can access it via https://www.vigiaccess.org/ to retrieve all ADR reports where vutrisiran is listed as the primary suspected drug. VigiAccess continuously updates treatment reports submitted by healthcare professionals and patients. The system employs automated algorithms to identify and remove duplicate reports. Additionally, it structures and categorizes the reports according to the Medical Dictionary for Regulatory Activities (MedDRA) hierarchy. Finally, the platform presents integrated statistical data based on the processed information. The characteristics of the ADR reports obtained from this database include the year of reporting, gender, age, and continental distribution. In the publicly accessible FAERS database, data are derived from ADR reports submitted to the FDA by healthcare professionals, consumers, and pharmacists. Although vutrisiran was first approved in June 2022, the FAERS database has been publicly available since the first quarter of 2004, with updates released quarterly. To ensure a comprehensive and unbiased data collection process, we extracted data covering the entire available period of the FAERS database (from Q1 2004 to Q3 2024; a total of 83 quarters), rather than restricting the search to the drug’s marketing period.

The data involved in this research come from public databases and do not involve human research, so ethical approval is not applicable.

### Data management and study design

Regarding the data derived from the FAERS database, the data management process includes removing duplicate reports and standardizing ADR terminology. Duplicate reports are processed according to FDA recommendations. Specifically, select the PRIMARYID, CASEID, and FDA_DT fields from the DEMO table, sort by CASEID, FDA_DT, and PRIMARYID, and for reports with the same CASEID, retain the one with the largest FDA_DT value; for those with the same CASEID and FDA_DT, retain the one with the largest PRIMARYID value. The standardization of ADR terminology is completed using the MedDRA dictionary version 27.1 to enhance the reliability of subsequent statistical analyses and their results.

### Statistical analysis

The descriptive research section is used to describe the characteristics of ADR reports with vutrisiran as the main suspected drug. The other sections use four disproportionality analysis methods and disproportionality approach Bayesian information component to detect signals of adverse reactions related to vutrisiran. Disproportionality analysis is a statistical technique used to detect potential associations between drugs and ADRs. The core principle of this method is to identify possible signals (i.e., potential drug safety issues) by comparing the observed frequency of ADR combinations with the expected frequency. For example, this can be achieved by calculating and comparing the frequencies of ADRs in exposed and non-exposed groups using a two-by-two contingency table. If the frequency of ADRs in the exposed group is higher than that in the non-exposed group, a positive signal is inferred, indicating a potential association between the drug and the ADR. Additionally, the strength of the signal can be assessed based on the magnitude of the positive signal.

The four disproportionality analysis methods are reporting odds ratio (ROR), [16] Bayesian confidence propagation neural network (BCPNN), [17] proportional reporting ratio (PRR), [18] and multi-item gamma Poisson shrinker (MGPS). [15] ADRs are determined to be potential ADRs of the drug through the combined use of the four disproportionality analysis methods. The detailed two-by-two contingency table is in the S1 Table in S1 File. Meanwhile, the formulas and thresholds of the disproportionality analysis methods are described in S2 Table in S1 File. To address the potential inflation of false-positive findings arising from multiple statistical comparisons across preferred terms (PTs), P-values derived from the chi-square statistics were adjusted using the Benjamini–Hochberg procedure to control the false discovery rate (FDR). Adjusted P-values (FDR < 0.05) were considered statistically significant. And PT events that represent medical procedures or end-stage therapeutic interventions (such as organ transplantation or device implantation), if identified, should be interpreted separately from typical adverse drug reactions because such events may reflect disease progression or clinical management decisions rather than direct drug-induced toxicity.

In addition, we conducted simulation analyses on the relevant data from the FAERS database, including Weibull distribution modeling and the cumulative incidence rate of ADRs. The Weibull distribution is used to simulate the change in the incidence of ADRs over time. The occurrence time of ADRs related to vutrisiran is defined as the time difference between the occurrence time of the ADR (as reported in the DEMO file) and the date of medication (as recorded in the THER file). For the time-to-onset analysis, reports with missing or incomplete therapy start dates, as well as those yielding negative or implausible extreme onset times, were considered unreliable and therefore excluded from the analysis. This study uses SAS 9.4 for statistical analysis.

## Results

### Descriptive analysis

The study included 721 ADR reports from the WHO-VigiAccess database and 732 ADR reports from the FAERS database. For the VigiAccess data, the reporting years were primarily 2023 (34.95%) and 2024 (64.49%), with a small portion from 2019 and 2022 (0.56%). As for the FAERS data, the earliest reports date back to 2022, with the majority also concentrated in 2023(44.67%) and 2024(52.87%). Among the WHO-VigiAccess database’s reports, 55 cases (7.63%) were male, and 41 cases (5.69%) were female. The majority of reports (86.69%), however, did not specify the patient’s gender. There is no significant gender difference. Likewise, most reports (91.68%) did not provide information on the patient’s age. Among those with specified ages, patients ranged from 18 to 75 years old. The data in the FAERS do not have detailed information about age and gender. It should be noted that a substantial proportion of demographic information, including age and gender, was missing from the dataset. However, as the signal detection analysis is driven by the distribution of adverse reaction reports rather than demographic variables, this missing information does not influence the calculation or interpretation of safety signals.

The vast majority of these reports originated from the Americas (85.58%), while 103 cases (14.29%) were from Europe, and only one case was reported in Asia. The data from the FAERS database exhibited a similar distribution, with the majority of reports originating from Americas (83.33%). In addition, relevant information about the reporting country and the time of adverse reactions occurring are provided in the FAERS. Data show that most of the reports from FAERS are from the United States, with 607 cases (82.92%). In addition, the FAERS database provides information on case seriousness. Among the reported cases, 594 (81.15%) were classified as serious. Specifically, 5 cases (0.68%) were life-threatening, 268 (36.61%) required hospitalization, 10 (1.37%) resulted in disability, 146 (19.95%) resulted in death, and 234 (31.97%) were associated with other medically important conditions. As serious outcomes can involve multiple categories, the sum of individual counts does not equal the total number of serious reports. In contrast, the WHO-VigiAccess database does not provide information on the severity or clinical outcomes of individual cases, and therefore these data were unavailable for analysis. Further details are presented in S3 Table in S1 File.

### Distribution of ADRs at the system organ class(SOC)level

**Fig 1:**
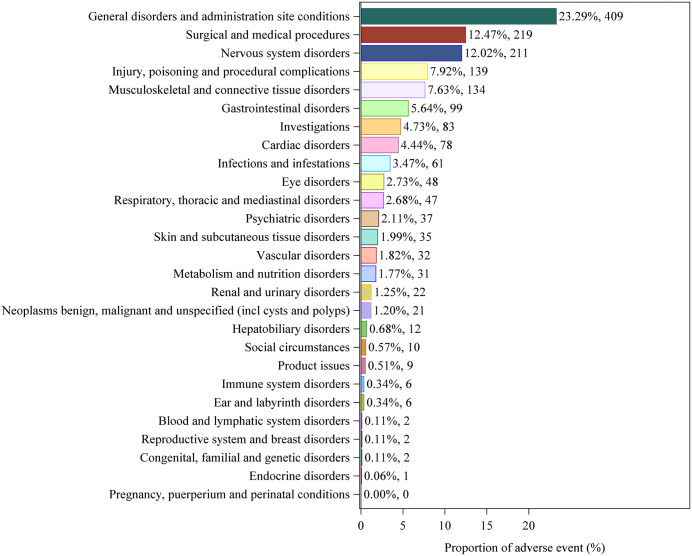
Distribution of ADRs in VigiAccess data at SOC level. Figure legend: This figure presents the proportional distribution of ADRs reports associated with vutrisiran at the MedDRA SOC level. A total of 1756 reports were included. The proportion for each SOC was calculated as the number of reports within that SOC divided by the total number of ADR reports, expressed as a percentage. The numbers shown on each bar indicate the corresponding percentage and absolute count of reports. Data were extracted from the VigiAccess. Abbreviation: ADR: Adverse Drug Reaction; MedDRA: Medical Dictionary for Regulatory Activities; SOC: System Organ Class.

**Fig 2:**
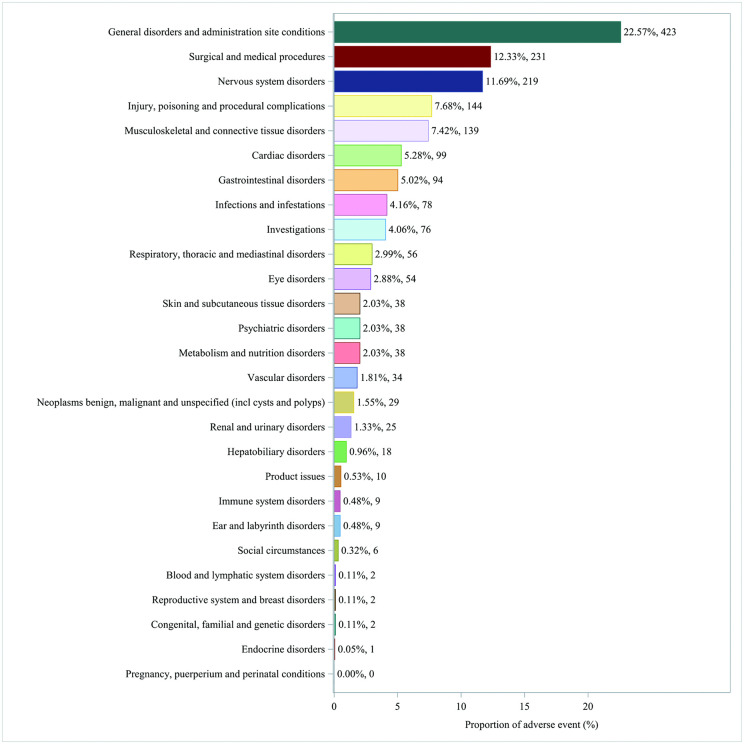
Distribution of ADRs in FAERS data at SOC level. Figure legend: This figure presents the proportional distribution of ADRs reports associated with vutrisiran at the MedDRA SOC level. A total of 1874 reports were included. The proportion for each SOC was calculated as the number of reports within that SOC divided by the total number of ADR reports, expressed as a percentage. The numbers shown on each bar indicate the corresponding percentage and absolute count of reports. Data were extracted from the FAERS. Abbreviation: ADR: Adverse Drug Reaction; MedDRA: Medical Dictionary for Regulatory Activities; SOC: System Organ Class; FAERS: Food and Drug Administration’s Adverse Event Reporting System.

Among the 27 SOCs, this study detected positive signals that met the requirements in 26 SOCs, with detailed information available in S4 Table in S1 File (WHO-VigiAccess database) and S5 Table in S1 File (FAERS database). The data from both databases showed the highest reporting rates in the SOC category of general disorders and administration site conditions. Additionally, the top five types of ADRs associated with vutrisiran at the SOC level were identified as follows: general disorders and administration site conditions, surgical and medical procedures, nervous system disorders, injury, poisoning and procedural complications and musculoskeletal and connective tissue disorders. In the ADR reports at the SOC level, both databases recorded three categories (general disorders and administration site conditions, surgical and medical procedures, and nervous system disorders) with incidence rates exceeding 10%. The distribution of ADRs at the SOC level is shown in Fig 1 and Fig 2.

### Distribution of ADRs at the preferred term(PT)level

**Fig 3:**
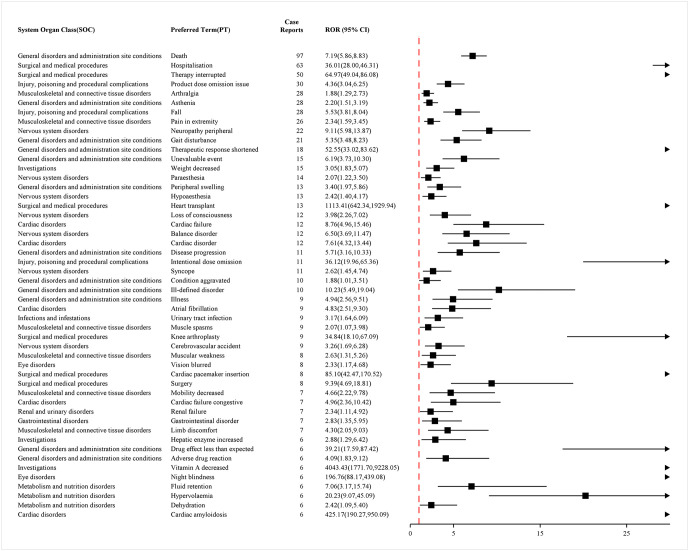
Top 50 positive signals of ADRs at PT level ranked by frequency of VigiAccess data. Figure legend: Forest plot showing RORs and corresponding 95% CIs for selected PTs identified in association with vutrisiran. Disproportionality analysis was conducted using the ROR, calculated by comparing the reporting frequency of each PT for vutrisiran with that for all other drugs in the VigiAccess database during the same study period. A positive signal was defined as a lower limit of the 95% CI greater than 1.0. Squares represent point estimates of the ROR, and horizontal lines indicate 95% CIs. The vertical dashed line denotes the null value (ROR = 1). PTs are grouped according to their corresponding SOC. Only PTs meeting predefined inclusion criteria are displayed. Abbreviation: ADR: Adverse Drug Reaction; ROR: Reporting Odds Ratio; CI: Confidence Intervals; PT: Preferred Term; SOC: System Organ Class.

**Fig 4:**
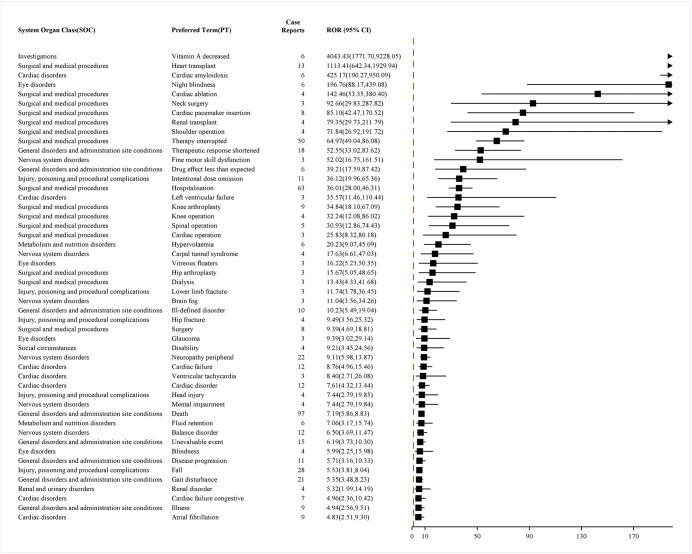
Top 50 positive signals of ADRs at PT level ranked by ROR of VigiAccess data. Figure legend: Forest plot showing RORs and corresponding 95% CIs for selected PTs identified in association with vutrisiran. Disproportionality analysis was conducted using the ROR, calculated by comparing the reporting frequency of each PT for vutrisiran with that for all other drugs in the VigiAccess database during the same study period. A positive signal was defined as a lower limit of the 95% CI greater than 1.0. Squares represent point estimates of the ROR, and horizontal lines indicate 95% CIs. The vertical dashed line denotes the null value (ROR = 1). PTs are grouped according to their corresponding SOC. Only PTs meeting predefined inclusion criteria are displayed. Abbreviation: ADR: Adverse Drug Reaction; ROR: Reporting Odds Ratio; CI: Confidence Intervals; PT: Preferred Term; SOC: System Organ Class.

**Fig 5:**
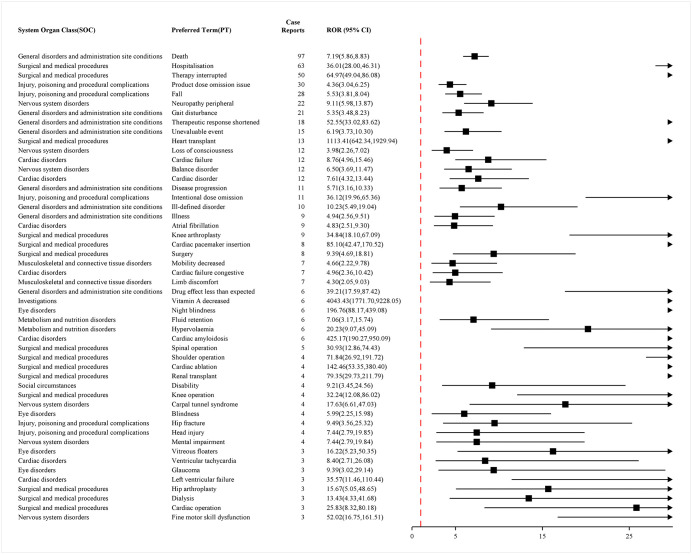
Top 50 ADRs that were positive through four methods ranked by frequency of VigiAccess data. Figure legend: Forest plot showing RORs and corresponding 95% CIs for selected PTs identified in association with vutrisiran, arranged in descending order of reporting frequency. Signals are detected when all the following criteria are meta ≥ 3, PRR ≥ 2 and Chi-Square ≥ 4, lower limit of 95% CI of ROR > 1, IC025 > 0, EBGM05 > 2. Squares represent point estimates of the ROR, and horizontal lines indicate 95% CIs. The vertical dashed line denotes the null value (ROR = 1). Only PTs meeting predefined inclusion criteria are displayed. Abbreviation: ADR: Adverse Drug Reaction; ROR: Reporting Odds Ratio; CI: Confidence Intervals; PT: Preferred Term; PRR: Proportional Reporting Ratio; EBGM: Empirical Bayes Geometric Mean.

**Fig 6:**
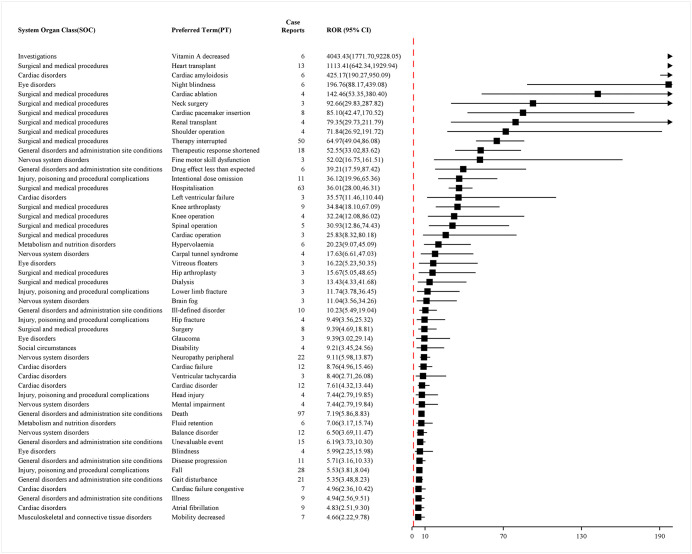
Top 50 ADRs that were positive through four methods ranked by ROR of VigiAccess data. Figure legend: Forest plot showing RORs and corresponding 95% CIs for selected PTs identified in association with vutrisiran, arranged in descending order of ROR. Signals are detected when all the following criteria are meta ≥ 3, PRR ≥ 2 and Chi-Square ≥ 4, lower limit of 95% CI of ROR > 1, IC025 > 0, EBGM05 > 2. Squares represent point estimates of the ROR, and horizontal lines indicate 95% CIs. The vertical dashed line denotes the null value (ROR = 1). Only PTs meeting predefined inclusion criteria are displayed. Abbreviation: ADR: Adverse Drug Reaction; ROR: Reporting Odds Ratio; CI: Confidence Intervals; PT: Preferred Term; PRR: Proportional Reporting Ratio; EBGM: Empirical Bayes Geometric Mean.

By using research methods to calculate the ROR values of various PTs, we identified PTs with positive signals in the ROR method. The top 50 PTs, ranked by frequency and signal strength, are presented in Fig 3 and Fig 4, respectively. To enhance the accuracy of the analysis, we analyzed ADR report data using a combination of four methods. A total of 53 PTs were screened out. The top 50 PTs, identified by the presence of positive signals across all four methods and ranked by frequency and signal strength, are presented in Fig 5 and Fig 6. Detailed results of this analysis can be found in S6 Table and S7 Table in S1 File. The positive signal situation of ADRs at the PT level is also evaluated.

**Fig 7:**
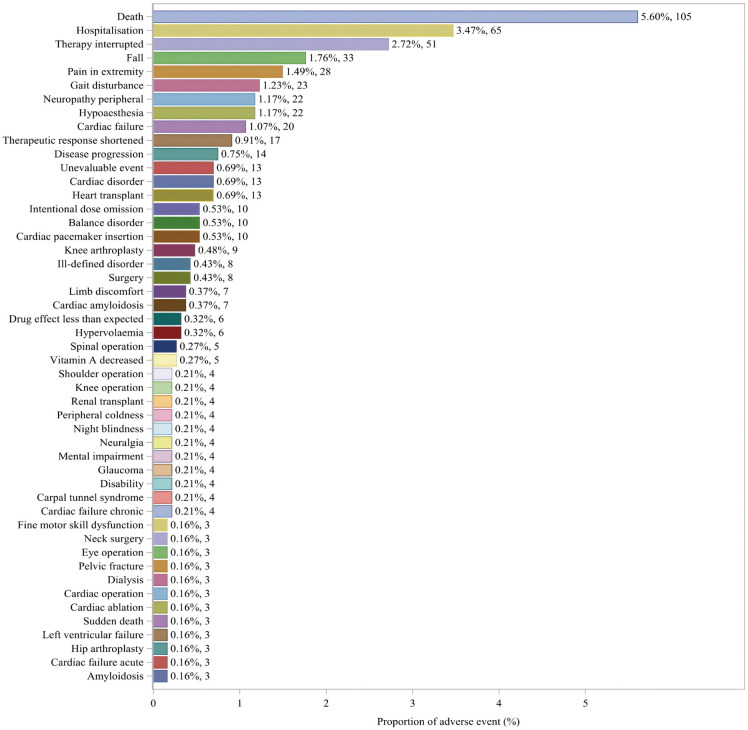
Top 49 positive signals of ADRs at PT level ranked by frequency of FAERS data. Figure legend: This figure presents the proportional distribution of ADRs reports associated with vutrisiran at the PT level. Signals are detected when all the following criteria are meta ≥ 3, PRR ≥ 2 and Chi-Square ≥ 4, lower limit of 95% CI of ROR > 1, IC025 > 0, EBGM05 > 2. Only PTs meeting predefined inclusion criteria are displayed. Abbreviation: ADR: Adverse Drug Reaction; ROR: Reporting Odds Ratio; CI: Confidence Intervals; PT: Preferred Term; PRR: Proportional Reporting Ratio; EBGM: Empirical Bayes Geometric Mean.

**Fig 8:**
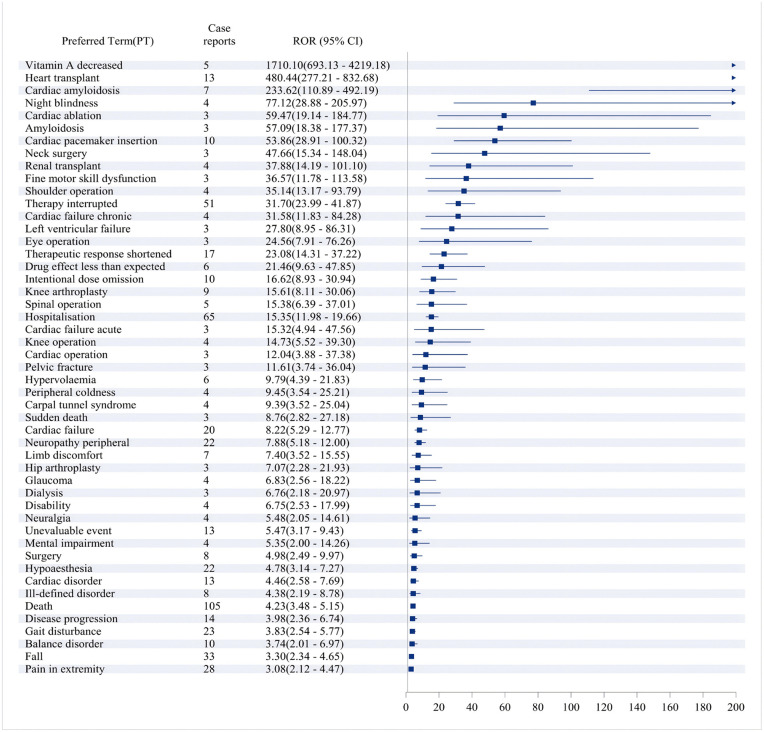
Top 49 positive signals of ADRs at PT level ranked by ROR of FAERS data. Figure legend: Forest plot showing RORs and corresponding 95% CIs for selected PTs identified in association with vutrisiran, arranged in descending order of ROR. Signals are detected when all the following criteria are meta ≥ 3, PRR ≥ 2 and Chi-Square ≥ 4, lower limit of 95% CI of ROR > 1, IC025 > 0, EBGM05 > 2. Squares represent point estimates of the ROR, and horizontal lines indicate 95% CIs. The vertical dashed line denotes the null value (ROR = 1). Only PTs meeting predefined inclusion criteria are displayed. Abbreviation: ADR: Adverse Drug Reaction; ROR: Reporting Odds Ratio; CI: Confidence Intervals; PT: Preferred Term; PRR: Proportional Reporting Ratio; EBGM: Empirical Bayes Geometric Mean.

A similar analysis was conducted on data from the FAERS database. To obtain more accurate and reliable results that are comparable with the aforementioned analysis, a disproportionality analysis was performed, yielding Fig 7 and Fig 8. A total of 49 PTs were screened out. These figures display the frequency-ranked and ROR-ranked results of PTs with positive signals, respectively. Detailed results of this analysis can be found in S8 Table in S1 File.

After applying the Benjamini–Hochberg correction for multiple testing, all PTs that met the predefined signal detection criteria remained statistically significant (FDR < 0.05). This indicates that the detected adverse event signals are robust and unlikely to have occurred by chance, suggesting a high level of confidence in the association between vutrisiran and these reported events.

The listed performance is the preferred choice within SOC. In addition to the currently known adverse reactions related to vutrisiran, there are: injection site reactions, such as redness, pain, or swelling at the injection site; systemic reactions like fatigue and myalgia; pain in extremity; and potential risks associated with reduced vitamin A levels. By comparing the analysis results from the aforementioned two databases, we detected potential positive signals not previously reported in the drug’s prescribing information, such as heart transplant, cardiac amyloidosis, night blindness, pelvic fractures, cardiac ablation, cardiac pacemaker insertion, neck surgery, renal transplantation, fine motor skill dysfunction, and shoulder operation, among others.

### Time to onset and weibull distribution analysis of ADRs

**Fig 9:**
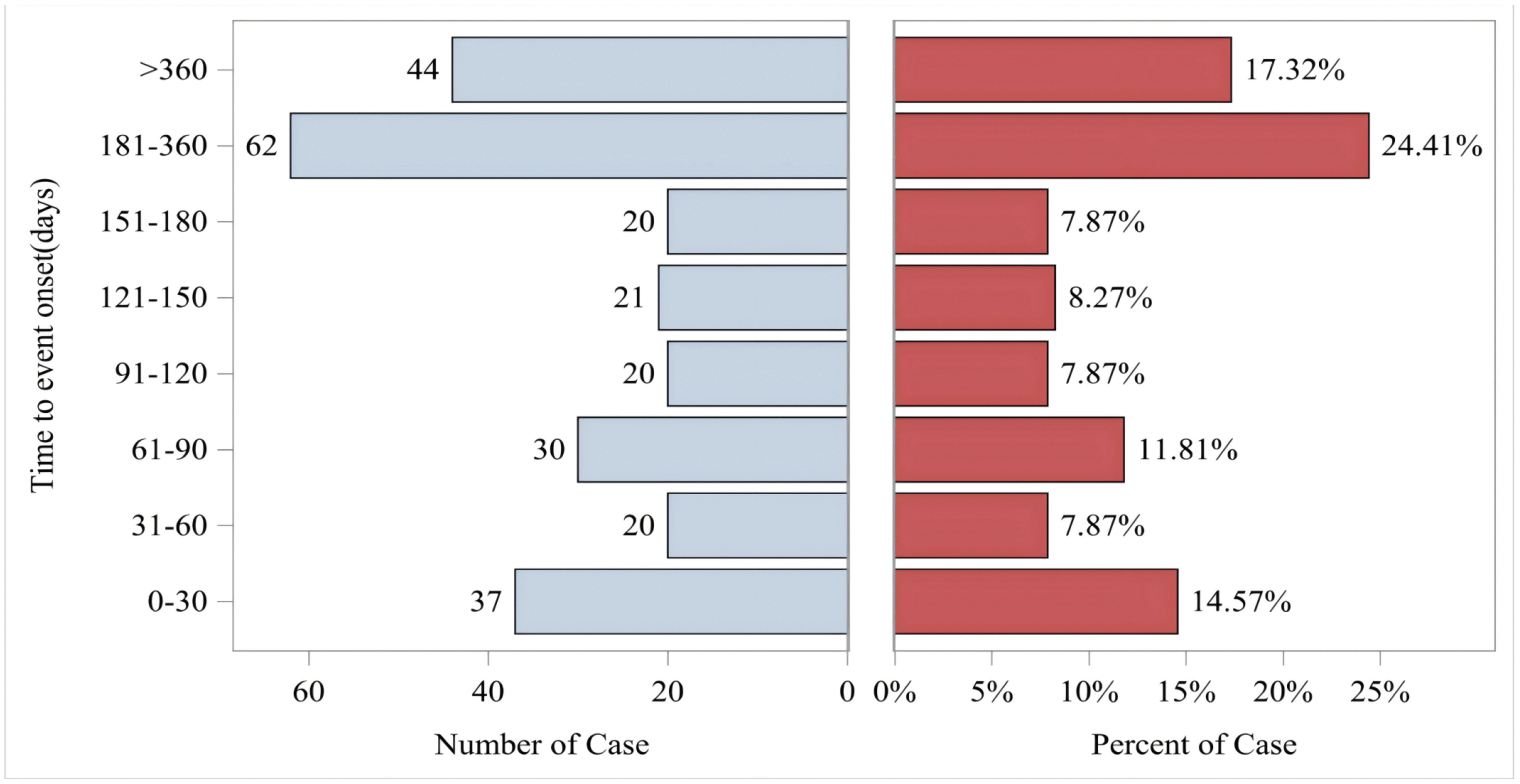
Time to event report distribution of ADR reports. Figure legend: Distribution of time to event following drug initiation. Time to event was defined as the interval between the reported start date of drug administration and the onset date of the adverse event. In the figure, the horizontal axis represents the number of cases and the percentage of cases, respectively, while the vertical axis represents the time to event onset (days).

In addition to the disproportionality analysis conducted on the data from the two databases mentioned above, we also performed Weibull distribution analysis and cumulative incidence simulation on the data from the FAERS database. The occurrence time of ADRs for vutrisiran is defined as the time difference between the occurrence time point of the ADR (reported in the DEMO file) and the medication date (recorded in the THER file). It can be seen from Fig 9 that the proportion of ADRs is quite large within 5 months after medication, and the proportion occurring from 5 months to 1 year is even greater than before. ADRs potentially related to vutrisiran appeared to occur predominantly within the first year following treatment.

**Fig 10:**
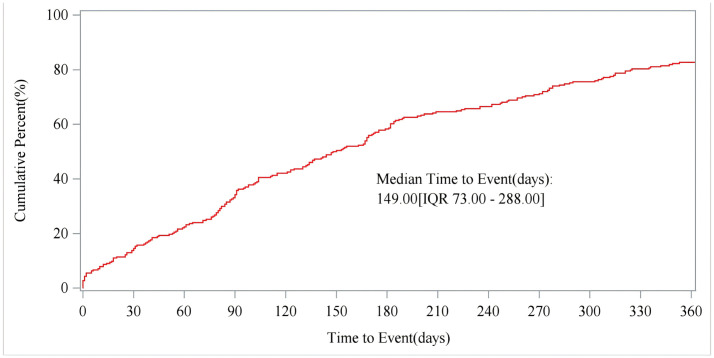
Cumulative incidence of ADRs. Figure legend: Cumulative incidence of ADRs modeled using Weibull distribution. Shape (β) parameter was used to characterize temporal patterns of adverse event occurrence following drug exposure, distinguishing early-onset and late-onset risk profiles among drugs.

The Weibull distribution fitting further supported the temporal characteristics of adverse reaction onset. The estimated shape parameter (β = 1.19) was slightly greater than 1, suggesting a modest increase in the occurrence rate of adverse reactions over time. This indicates that the risk of developing adverse events may accumulate gradually rather than appearing immediately after treatment initiation. The estimated scale parameter (η = 202.16 days) implies that the characteristic time for event onset is approximately six to seven months after drug exposure. Together, these findings corroborate our observation that the majority of adverse reactions tend to occur within the first year following the initiation of vutrisiran therapy, reflecting a delayed-onset safety profile consistent with the pharmacological nature of the drug. The specific distribution of the occurrence time of ADRs is shown in Fig 9. The cumulative incidence curve of ADRs is shown in Fig 10.

**Fig 11:**
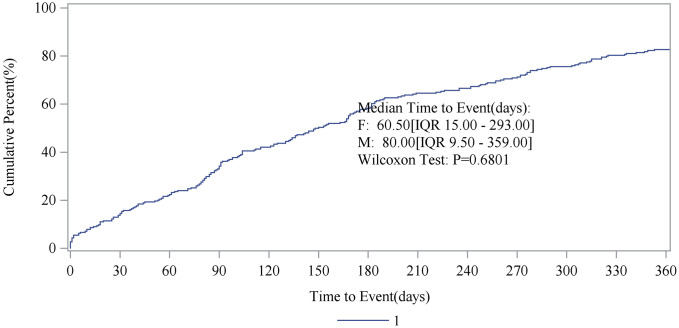
The relationship between gender and the cumulative incidence of ADRs. Cumulative incidence of ADRs stratified by gender. The cumulative percentage of patients experiencing ADRs is plotted against time to event. The x‑axis denotes time from drug initiation, and the y‑axis shows the cumulative percentage of patients with ADRs.

The study also stratified the data by gender to examine differences in the cumulative incidence of ADRs following drug use. Fig 11 presents the findings. The median time to event was 60.50 days (IQR: 15.00–293.00) for females and 80.00 days (IQR: 9.50–359.00) for males. The Wilcoxon test yielded a P-value of 0.6801. This indicates that while there are differences in the cumulative incidence of ADRs between genders, these differences are not statistically significant. To some extent, it can be concluded that the cumulative incidence of ADRs is not associated with gender.

## Discussion

SRS are widely used for evaluating the safety of suspected ADRs associated with marketed drugs. Since the data in these systems are derived from reports by patients following drug use, they provide a more accurate reflection of real-world drug safety. Although pre-marketing drug trials are rigorous, they are conducted under a set of constraints, such as specific regions and environments, which may limit the understanding of drug safety. In contrast, utilizing SRS offers a more comprehensive insight into the real-world safety of drug use. Commonly used databases for real-world drug safety signal research include the FAERS, WHO-VigiBase, and the EudraVigilance Data Analysis System (EVDAS). [19] The data for this study were sourced from WHO-VigiAccess and FAERS.

By applying relevant methods to analyze the ADR data, in addition to the adverse reactions already confirmed for vutrisiran, including injection site reactions, such as redness, pain, or swelling at the injection site; systemic reactions like fatigue and myalgia; pain in extremity; and potential risks associated with reduced vitamin A levels, we observed positive signals showing a strong potential association with the drug such as: surgical and medical procedures (heart transplant, cardiac ablation, cardiac pacemaker insertion, neck surgery, renal transplant, shoulder operation), cardiac disorders (cardiac amyloidosis), eye disorders (night blindness), and nervous system disorders (fine motor skill dysfunction). Therefore, further research on the safety and adverse reactions of vutrisiran is needed, especially regarding the mitigation and prevention of adverse reactions occurring within one year after treatment with vutrisiran. It should be emphasized that several infrequent signals identified in this study—particularly those related to organ transplantation and surgical or interventional procedures—should be interpreted with caution. In spontaneous reporting systems such as FAERS, these events are more likely to represent indicators of underlying disease severity, comorbid complications of transthyretin-mediated amyloidosis, or downstream clinical management decisions, rather than direct adverse drug reactions attributable to vutrisiran. As such, their detection as statistical signals does not imply causality.

Vitamin A deficiency is a common adverse reaction of vutrisiran. In a study trial, patients were randomly assigned in a 3:1 ratio to receive subcutaneous injections of 25 mg AMVUTTRA (vutrisiran, N = 122) every three months or intravenous injections of 0.3 mg/kg patisiran (N = 42) as a control group. Among the 122 patients receiving AMVUTTRA treatment, 7% experienced vitamin A deficiency, and based on the intake and baseline levels of vitamin A in the trial, it can be considered that in some cases, vitamin A deficiency can be regarded as an adverse reaction. [1] This trial result is consistent with our analysis.

At the same time, we know that vitamin A deficiency often leads to night blindness. [20,21] Coincidentally, our analysis detected a potential association between vutrisiran and night blindness, which is consistent with our analysis findings. Night blindness severely reduces patients’ vision at night, significantly affecting their quality of life. [21,22] Given these findings, particular attention should be paid to the close association between vutrisiran use and the occurrence of night blindness. Although a definitive causal relationship has not been established, the correlation appears noteworthy and clinically relevant. The development of night blindness in patients treated with vutrisiran may result from vitamin A deficiency secondary to its pharmacological effects, or possibly from other underlying mechanisms yet to be clarified. Further basic and clinical studies are needed to elucidate the exact biological pathways involved in this phenomenon. Therefore, in clinical practice, patients receiving vutrisiran should be informed of this potential risk, and preventive strategies—such as concurrent vitamin A supplementation—may be considered. If symptoms of night blindness arise, timely evaluation and treatment are essential. In conclusion, both clinicians and patients should maintain vigilance toward visual disturbances potentially associated with vutrisiran therapy, and continued research into their mechanisms remains of substantial importance.

Our findings suggest a notable association between vutrisiran use and bone-related adverse events, particularly pelvic fractures. Although a causal relationship cannot yet be established, this potential link warrants careful attention. Vitamin A deficiency is a well-documented and relatively common adverse reaction to vutrisiran therapy, and accumulating evidence indicates that disturbances in vitamin A metabolism can profoundly influence bone health. Retinol, the active form of vitamin A, plays a crucial role in maintaining bone remodeling and mineralization. Insufficient retinol levels have been associated with reduced osteoblast activity, increased bone fragility, and a higher incidence of fractures, including those of the hip and pelvis. [23,24] Given these connections, it is plausible that the observed pelvic fractures in vutrisiran-treated patients may, at least in part, relate to altered vitamin A homeostasis. However, ATTR patients frequently present with advanced age, multisystem involvement, and substantial comorbidity burdens, and are often exposed to multiple concomitant medications, all of which may independently contribute to the development of gout or skeletal complications. Therefore, confounding by underlying disease severity, comorbid conditions, and polypharmacy is highly likely. Taken together, while the observed associations warrant clinical awareness and further investigation, causal inference cannot be drawn from spontaneous reporting data alone. Future studies incorporating controlled designs and detailed clinical information are needed to clarify whether these signals reflect drug-related effects, disease-related processes, or background risks in this patient population.

Our analysis indicates a close association between vutrisiran use and cardiac-related adverse events, particularly cardiac pacemaker insertion and cardiac disorder. Although a causal relationship cannot yet be confirmed, this association appears clinically meaningful. In the HELIOS-B trial, the incidence of severe atrial fibrillation was reported to be 8% (26 cases) in the vutrisiran group, higher than the 6% (20 cases) observed in the control group. [9] Another study found that 24.6% of patients treated with vutrisiran experienced arrhythmias, with supraventricular arrhythmias and cardiac conduction disorders being the most frequent manifestations. [25] Moreover, in cases of vutrisiran overdose, the risk of atrioventricular (AV) block and complete AV block was notably increased. [1] These findings collectively suggest that heart diseases such as arrhythmia may have a strong correlation with vutrisiran. Cardiac pacemaker insertion, a therapeutic approach primarily used to manage severe bradyarrhythmias and conduction abnormalities, often serves as a clinical response to such cardiac complications. [26] However, these cardiac-related adverse events are also clear clinical manifestations of cardiac involvement in the middle and late stages of ATTR. Therefore, the positive signals may also reflect disease progression or routine clinical management rather than primary adverse drug reactions.In this context, the occurrence of these events in vutrisiran-treated patients should be interpreted more as a commonly reported clinical outcome in a population with severe underlying heart disease rather than evidence of a direct pharmacological effect. Although a temporal association between vutrisiran therapy and arrhythmic events was observed in some reports, the limitations inherent to spontaneous reporting data preclude causal inference. Nevertheless, from a clinical perspective, continued vigilance for cardiac conduction abnormalities and rhythm disturbances remains warranted in patients receiving vutrisiran, with appropriate monitoring and management strategies implemented as part of standard care.

Signals related to renal transplantation was identified in the present analysis; however, their interpretation requires particular caution. In patients with ATTR, renal transplantation typically reflects advanced disease stages, complex comorbid conditions, and major clinical decision-making processes rather than a direct adverse effect of short- or medium-term drug exposure. The observed renal transplant–related reports may therefore be influenced by multiple interrelated factors, including underlying disease severity and clinical management trajectories. In addition, intercurrent complications such as severe or recurrent urinary tract infections, which may progress to acute kidney injury, could plausibly accelerate renal function deterioration in susceptible individuals and indirectly contribute to the need for renal replacement therapies, including transplantation. In a previous clinical trial, investigators attributed two serious adverse events to vutrisiran—one urinary tract infection and one case of dyslipidemia—representing 1.6% of participants. [27]Nevertheless, given the inherent limitations of SRS, these observations should not be interpreted as evidence of a definitive mechanistic association between vutrisiran and renal transplantation. Instead, such procedure-related signals are more appropriately viewed as contextual clinical outcomes reflecting patient severity and healthcare utilization. From a clinical perspective, routine monitoring of renal function and urinary complications remains prudent in this patient population, while further well-designed clinical studies are warranted to clarify the relative contributions of drug exposure, disease progression, and intercurrent infections to adverse renal outcomes.

We also identified signals such as therapy interruption, peripheral neuropathy and even mental impairment in patients treated with vutrisiran, which may reflect a potential association with the drug. It should be noted, however, that nervous‑system manifestations are also common in the transthyretin-mediated amyloidosis itself, and therefore a direct causal attribution to the drug cannot be established at this stage. Therefore, more specialized mechanistic studies are needed to explore the relationship between vutrisiran and neurological abnormalities.

The analysis conducted a temporal analysis of the data and a Weibull distribution analysis, predicting the timing and incidence of adverse reactions primarily associated with vutrisiran. The results suggest that we should pay attention to and monitor the occurrence of these ADRs within one year after drug treatment. At the same time, it is important to emphasize the temporal monitoring of drug adverse reactions, as this is significant for our early prevention and management of adverse reactions and for mitigating their impact on patients’ quality of life. However, this finding should be interpreted with caution because of the inherent limitations of spontaneously reported data.

Of course, this analytical study also has certain limitations. Due to various influencing factors, the data obtained in this study are not entirely comprehensive. For example, in both databases analyzed, gender information is largely unavailable, which may introduce bias in analyses requiring gender stratification and consequently affect the accuracy of the findings. To mitigate this limitation, expanding the data volume as much as possible could help reduce potential errors. And the description of patients’ age distribution in the manuscript also may not be entirely accurate because part of the age information is missing in datasets. Therefore, additional and more comprehensive data are needed to provide a more reliable characterization of the age distribution.

In addition, a substantial portion of reports lacked complete time-to-onset information, which may restrict the accuracy of temporal pattern analysis. The Weibull distribution was therefore applied only to cases with available onset data. Since these missing data were likely attributable to incomplete spontaneous reporting rather than systematic bias, the resulting estimates still provide a reasonable approximation of the overall time-to-onset pattern. Nevertheless, this limitation should be considered when interpreting the temporal trend of adverse reaction occurrence.

Moreover, the majority of reports originate from the Americas (FAERS: 83.06%; WHO-VigiAccess: 85.58%), while only a small fraction comes from Asia. This geographical imbalance may be attributed to the fact that vutrisiran is currently approved and marketed mainly in the United States and Europe, whereas it has not yet been introduced in most Asian countries. Another possible reason is that many Asian nations are still developing economies and may lack sufficient medical and financial resources to access advanced therapies like vutrisiran for rare diseases. Consequently, the present findings, derived primarily from U.S. data, may not accurately represent the situation in other regions such as Asia or Europe, potentially introducing a certain degree of bias. Nevertheless, real-world safety studies of vutrisiran remain crucial, as they can provide valuable evidence to support its broader clinical application and global accessibility.

In addition, the data utilized in this study were derived from spontaneous reports submitted by healthcare professionals, consumers, and pharmacists to the FAERS database. Such reports are subject to inherent limitations, including underreporting, reporting bias, and incomplete or inaccurate information. These issues may obscure the true incidence or causality of adverse events, thereby influencing the reliability of the conclusions. Furthermore, from a methodological perspective, this study mainly relied on the descriptive analysis of spontaneous reporting data, focusing on the reported adverse events, the drug itself, and its approved indications. The lack of validation from large-scale, controlled clinical trials limits the ability to infer causal relationships and may lead to over- or underestimation of safety signals.

Therefore, the safety signals identified in this study should be interpreted cautiously, considering the above-mentioned limitations of data quality and regional coverage. Future research should aim to incorporate larger and more diverse datasets covering multiple geographic regions to minimize potential bias and improve representativeness. In parallel, well-designed clinical trials and post-marketing surveillance studies are warranted to further substantiate and validate the real-world safety profile of vutrisiran.

## Conclusion

In this analysis, we analyzed relevant data from WHO-VigiAccess and the FAERS database, using four disproportionality analysis methods. The results confirmed known adverse reactions such as vitamin A deficiency, and also detected some potential ADRs, including night blindness. Signals related to advanced cardiac or renal interventions were observed but likely reflect underlying disease severity rather than direct drug effects. These research and analysis results provide doctors with more safety information to consider the adverse reactions of this drug.

## Supporting information

S1 File This file contains Supplementary Tables S1–S8. S1 Table. Two-by-two contingency table for disproportionality analyses. S2 Table. Formulas and thresholds of disproportionality analysis methods. S3 Table. Characteristics of adverse drug reactions reports. S4 Table. Signal strength of ADEs at the System Organ Class (SOC) level in Vigiaccess database. S5 Table. Signal strength of ADEs at the System Organ Class (SOC) level in FAERS database. S6 Table. Signal strength of adverse drug reactions at the Preferred Term (PT) level ranked by ROR. S7 Table. Signal strength of adverse drug reactions at the Preferred Term (PT) level ranked by Reports. S8 Table. Signal strength of adverse drug reactions at the Preferred Term (PT) level ranked by ROR.
(DOCX)
