# Supplementary material for: Assessing the real-world safety of vutrisiran for transthyretin-mediated amyloidosis with polyneuropathy: Based on WHO-VigiAccess and FAERS databases
Source: PLoS One. 2026 Apr 15;21(4):e0347417. doi: 10.1371/journal.pone.0347417 (PMC13082648; doi:10.1371/journal.pone.0347417)
Supplement: S1 File — S1 Table. Two-by-two contingency table for disproportionality analyses. S2 Table. Formulas and thresholds of disproportionality analysis methods. S3 Table. Characteristics of adverse drug reactions reports. S4 Table. Signal strength of ADEs at the System Organ Class (SOC) level in Vigiaccess database. S5 Table. Signal strength of ADEs at the System Organ Class (SOC) level in FAERS database. S6 Table. Signal strength of adverse drug reactions at the Preferred Term (PT) level ranked by ROR. S7 Table. Signal strength of adverse drug reactions at the Preferred Term (PT) level ranked by Reports. S8 Table. Signal strength of adverse drug reactions at the Preferred Term (PT) level ranked by ROR. (DOCX) [file pone.0347417.s001.docx]

**Supplementary Table S1** Two-by-two contingency table for disproportionality analyses

|  | **Target AEs** | **Other AEs** | **Total** |
| --- | --- | --- | --- |
| **Vutrisiran** | a | b | a +b |
| **Other Drugs** | c | d | c +d |
| **Total** | a +c | b +d | N=a + b +c +d |

Abbreviations: ADRs, adverse drug reactions; a, the number of reports containing target ADRs caused by vutrisiran; b, the number of reports containing other ADRs caused by vutrisiran; c, the number of reports containing target ADRs caused by other drugs; d, the number of reports containing other ADRs caused by other drugs; n, the total number of adverse drug reaction occurrences included in the background data analysis.

**Supplementary Table S2** Formulas and thresholds of disproportionality analysis methods

| Vutrisiran | Formula | Threshold |
| --- | --- | --- |
| ROR | 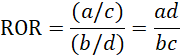  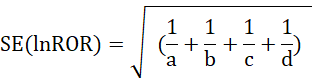  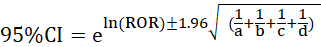 | a ≥ 3 and 95% CI (lower limit) > 1 suggests generation of 1 signal. |
| PRR | 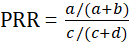  SE (ln PRR) =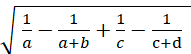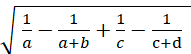  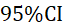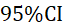=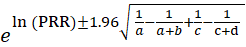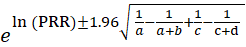  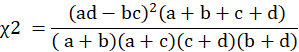 | Lower limit of the PRR confidence interval: a ≥ 3 and 95% CI (lower limit) > 1 suggests generation of 1 signal.  MHRA composite criterion method: a ≥ 3 and PRR value ≥ 2 and χ² ≥ 4 suggests generation of 1 signal |
| BCPNN | IC=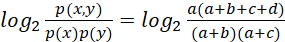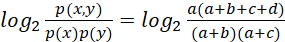  E(IC)=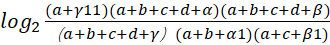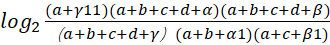  V(IC)=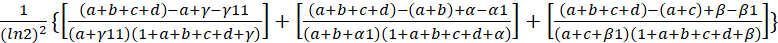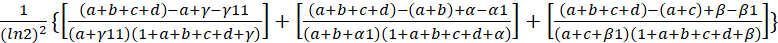  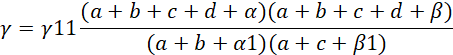  *IC-2SD=E(IC)-2*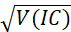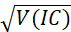  α1=β1=1；α=β=2；γ11=1 | The lower limit of the confidence interval (IC-2SD) is greater than 0, then 1 signal is prompted to be generated |
| MGPS | 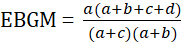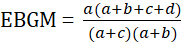  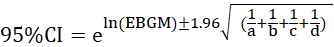 | EBGM05>2 prompts to generate 1 signal  EBGM05 indicates the lower limit of the EBGM 95% confidence interval |

Abbreviations: ROR, Reporting Odds Ratio; PRR, Proportional Reporting Ratio; BCPNN, Bayesian confidence propagation neural network; MGPS, Muti-item Gamma Poisson Shrinker; MHRA, Medicines and Healthcare Products Regulatory Agency. The results of the PRR lower confidence interval method for signal detection are almost the same as those of the ROR method, and the threshold of the MHRA composite standard method is commonly used for signal detection. In the summary report, the threshold of the MHRA composite standard method will be used as the threshold of the PRR method for signal detection.

**Supplementary Table S3** Characteristics of adverse drug reactions reports

| **Characteristics** | **n (%)** | |
| --- | --- | --- |
|  | **WHO-VigiAccess(n=721)** | **FAERS(n=732)** |
| Sex |  |  |
| Female | 41(5.69) |  |
| Male | 55(7.63) |  |
| Unknown | 625(86.69) | 732(100.0) |
| Age |  |  |
| 18 - 44 years | 7(0.97) |  |
| 45 - 64 years | 16(2.22) |  |
| 65 - 74 years | 19(2.64) |  |
| 75 years | 18(2.5) |  |
| Unknown | 661(91.68) | 732(100.0) |
| Continent |  |  |
| Americas | 617(85.58) | 610(83.33) |
| Asia | 1(0.14) | 53(7.24) |
| Europe | 103(14.29) | 69(9.43) |
| Report year |  |  |
| 2019 | 1(0.14) | 0(0.00) |
| 2022 | 3(0.42) | 18(2.46) |
| 2023 | 252(34.95) | 327(44.67) |
| 2024 | 465(64.49) | 387(52.87) |
| Report countries |  |  |
| United States of America |  | 607(82.92) |
| Japan |  | 53(7.24) |
| France |  | 33(4.51) |
| Germany |  | 15(2.05) |
| United Kingdom |  | 10(1.37) |
| Time of adverse drug reactions - date of medication (day) segments |  |  |
| 0-30d |  | 37(5.05) |
| 31-60d |  | 20(2.73) |
| 61-90d |  | 30(4.10) |
| 91-120d |  | 20(2.73) |
| 121-150d |  | 21(2.87) |
| 151-180d |  | 20(2.73) |
| 181-360d |  | 62(8.47) |
| ＞360 d |  | 44(6.01) |
| Missing or outlier (less than 0) (%) |  | 478(65.30) |
| Serious report |  |  |
| Serious (%) |  | 594(81.15) |
| Non-Serious (%) |  | 138(18.85) |
| Outcome |  |  |
| Life-Threatening (%) |  | 5(0.68) |
| Hospitalization - Initial or Prolonged (%) |  | 268(36.61) |
| Disability (%) |  | 10(1.37) |
| Death (%) |  | 146(19.95) |
| Congenital Anomaly (%) |  | 0(0.00) |
| Required Intervention to Prevent Permanent Impairment/Damage (%) |  | 0(0.00) |
| Other (%) |  | 234(31.97) |

Note 1: The date of adverse drug reactions collected by the database refers to the date when the patient first experienced an adverse drug reaction (without specifying the specific name of the adverse drug reaction).

Note 2: Outcomes are for the patient dimension, not specific adverse events. The same patient may have multiple outcome outcomes in the database, so the sum of the outcomes may not be 100%.

Note 3: Serious reports are for the patient dimension, not specific adverse events. Those with outcomes in the FAERS database are serious reports, and those with unfilled outcomes are non-serious reports.

**Supplementary Table S4** Signal strength of ADEs at the System Organ Class (SOC) level in Vigiaccess database

| **System Organ Class (SOC)** | **Case reports** | **ROR (95% CI)** | **PRR (95% CI)** | **Chi Square** | **IC (IC025)** | **EBGM (EBGM05)** |
| --- | --- | --- | --- | --- | --- | --- |
| General disorders and administration site conditions | 409 | 1.19(1.07,1.33) | 1.15(1.05,1.25) | 9.80 | 0.20(0.04) | 1.15(1.03) |
| Surgical and medical procedures | 219 | 17.44(15.14,20.09) | 15.39(13.60,17.42) | 2970.38 | 3.94(3.65) | 15.39(13.36) |
| Nervous system disorders | 211 | 1.20(1.04,1.38) | 1.17(1.03,1.33) | 6.01 | 0.23(0.02) | 1.17(1.02) |
| Injury, poisoning and procedural complications | 139 | 1.26(1.06,1.49) | 1.24(1.05,1.45) | 6.66 | 0.30(0.05) | 1.24(1.04) |
| Musculoskeletal and connective tissue disorders | 134 | 1.49(1.25,1.77) | 1.45(1.23,1.71) | 19.72 | 0.54(0.27) | 1.45(1.22) |
| Gastrointestinal disorders | 99 | 0.54(0.44,0.66) | 0.57(0.47,0.69) | 36.13 | -0.82(-1.11) | 0.57(0.46) |
| Investigations | 83 | 0.74(0.59,0.92) | 0.75(0.61,0.92) | 7.47 | -0.42(-0.74) | 0.75(0.60) |
| Cardiac disorders | 78 | 1.91(1.52,2.40) | 1.87(1.51,2.33) | 32.47 | 0.90(0.56) | 1.87(1.49) |
| Infections and infestations | 61 | 0.86(0.67,1.11) | 0.87(0.68,1.11) | 1.30 | -0.21(-0.58) | 0.87(0.67) |
| Eye disorders | 48 | 1.60(1.20,2.13) | 1.58(1.20,2.09) | 10.50 | 0.66(0.23) | 1.58(1.19) |
| Respiratory, thoracic and mediastinal disorders | 47 | 0.60(0.45,0.80) | 0.61(0.46,0.81) | 12.37 | -0.72(-1.13) | 0.61(0.46) |
| Psychiatric disorders | 37 | 0.43(0.31,0.60) | 0.44(0.32,0.61) | 27.00 | -1.17(-1.62) | 0.44(0.32) |
| Skin and subcutaneous tissue disorders | 35 | 0.21(0.15,0.29) | 0.23(0.16,0.31) | 102.00 | -2.15(-2.60) | 0.23(0.16) |
| Vascular disorders | 32 | 0.85(0.60,1.21) | 0.85(0.61,1.20) | 0.81 | -0.23(-0.73) | 0.85(0.60) |
| Metabolism and nutrition disorders | 31 | 0.99(0.69,1.41) | 0.99(0.70,1.40) | 0.01 | -0.02(-0.53) | 0.99(0.69) |
| Renal and urinary disorders | 22 | 0.78(0.51,1.19) | 0.78(0.52,1.19) | 1.35 | -0.35(-0.95) | 0.78(0.51) |
| Neoplasms benign, malignant and unspecified (incl cysts and polyps) | 21 | 0.86(0.56,1.32) | 0.86(0.56,1.31) | 0.51 | -0.22(-0.83) | 0.86(0.56) |
| Hepatobiliary disorders | 12 | 0.86(0.49,1.51) | 0.86(0.49,1.51) | 0.29 | -0.22(-1.01) | 0.86(0.49) |
| Social circumstances | 10 | 1.66(0.89,3.09) | 1.66(0.89,3.08) | 2.62 | 0.73(-0.23) | 1.66(0.89) |
| Product issues | 9 | 0.57(0.29,1.09) | 0.57(0.30,1.09) | 2.96 | -0.81(-1.67) | 0.57(0.30) |
| Ear and labyrinth disorders | 6 | 0.63(0.28,1.41) | 0.63(0.29,1.41) | 1.28 | -0.66(-1.67) | 0.63(0.28) |
| Immune system disorders | 6 | 0.27(0.12,0.60) | 0.27(0.12,0.60) | 11.84 | -1.88(-2.81) | 0.27(0.12) |
| Congenital, familial and genetic disorders | 2 | 0.67(0.17,2.68) | 0.67(0.17,2.68) | 0.33 | -0.58(-2.08) | 0.67(0.17) |
| Reproductive system and breast disorders | 2 | 0.11(0.03,0.42) | 0.11(0.03,0.43) | 15.10 | -3.23(-4.39) | 0.11(0.03) |
| Blood and lymphatic system disorders | 2 | 0.05(0.01,0.20) | 0.05(0.01,0.21) | 35.68 | -4.28(-5.40) | 0.05(0.01) |
| Endocrine disorders | 1 | 0.28(0.04,2.01) | 0.28(0.04,2.01) | 1.82 | -1.82(-3.22) | 0.28(0.04) |

Note: Ranked by case reports.

**Supplementary Table S5** Signal strength of ADEs at the System Organ Class (SOC) level in FAERS database

| **System Organ Class (SOC)** | **Case reports** | **ROR (95% CI)** | **PRR (95% CI)** | **Chi Square** | **IC (IC025)** | **EBGM (EBGM05)** |
| --- | --- | --- | --- | --- | --- | --- |
| General disorders and administration site conditions | 423 | 1.38(1.24,1.54) | 1.29(1.19,1.41) | 34.31 | 0.37(0.22) | 1.29(1.16) |
| Surgical and medical procedures | 231 | 10.33(9.00,11.85) | 9.18(8.14,10.36) | 1705.97 | 3.20(2.95) | 9.18(8.00) |
| Nervous system disorders | 219 | 1.42(1.24,1.64) | 1.37(1.21,1.56) | 24.35 | 0.46(0.25) | 1.37(1.19) |
| Injury, poisoning and procedural complications | 144 | 0.72(0.61,0.86) | 0.74(0.64,0.87) | 14.18 | -0.43(-0.67) | 0.74(0.63) |
| Musculoskeletal and connective tissue disorders | 139 | 1.47(1.23,1.74) | 1.43(1.22,1.68) | 19.12 | 0.52(0.26) | 1.43(1.20) |
| Cardiac disorders | 99 | 2.06(1.68,2.52) | 2.00(1.65,2.42) | 50.88 | 1.00(0.69) | 2.00(1.63) |
| Gastrointestinal disorders | 94 | 0.57(0.46,0.70) | 0.59(0.48,0.72) | 29.34 | -0.76(-1.06) | 0.59(0.48) |
| Infections and infestations | 78 | 0.79(0.63,0.99) | 0.80(0.64,0.99) | 4.34 | -0.33(-0.66) | 0.80(0.63) |
| Investigations | 76 | 0.65(0.51,0.81) | 0.66(0.53,0.82) | 14.23 | -0.60(-0.93) | 0.66(0.52) |
| Respiratory, thoracic and mediastinal disorders | 56 | 0.62(0.48,0.81) | 0.64(0.49,0.82) | 12.31 | -0.65(-1.03) | 0.64(0.49) |
| Eye disorders | 54 | 1.46(1.11,1.92) | 1.45(1.11,1.88) | 7.63 | 0.53(0.13) | 1.45(1.10) |
| Metabolism and nutrition disorders | 38 | 0.93(0.68,1.29) | 0.93(0.68,1.28) | 0.18 | -0.10(-0.56) | 0.93(0.68) |
| Psychiatric disorders | 38 | 0.35(0.25,0.48) | 0.36(0.26,0.49) | 46.18 | -1.48(-1.92) | 0.36(0.26) |
| Skin and subcutaneous tissue disorders | 38 | 0.36(0.26,0.50) | 0.38(0.28,0.52) | 41.34 | -1.41(-1.85) | 0.38(0.27) |
| Vascular disorders | 34 | 0.85(0.60,1.19) | 0.85(0.61,1.18) | 0.93 | -0.24(-0.72) | 0.85(0.60) |
| Neoplasms benign, malignant and unspecified (incl cysts and polyps) | 29 | 0.58(0.40,0.84) | 0.59(0.41,0.84) | 8.66 | -0.77(-1.28) | 0.59(0.41) |
| Renal and urinary disorders | 25 | 0.69(0.47,1.03) | 0.70(0.47,1.03) | 3.30 | -0.52(-1.07) | 0.70(0.47) |
| Hepatobiliary disorders | 18 | 1.05(0.66,1.67) | 1.05(0.66,1.66) | 0.04 | 0.07(-0.60) | 1.05(0.66) |
| Product issues | 10 | 0.33(0.18,0.61) | 0.33(0.18,0.61) | 13.75 | -1.60(-2.38) | 0.33(0.18) |
| Ear and labyrinth disorders | 9 | 1.11(0.57,2.13) | 1.11(0.58,2.12) | 0.09 | 0.14(-0.79) | 1.11(0.57) |
| Immune system disorders | 9 | 0.43(0.23,0.84) | 0.44(0.23,0.84) | 6.60 | -1.19(-2.03) | 0.44(0.23) |
| Social circumstances | 6 | 0.69(0.31,1.53) | 0.69(0.31,1.53) | 0.85 | -0.54(-1.56) | 0.69(0.31) |
| Congenital, familial and genetic disorders | 2 | 0.35(0.09,1.41) | 0.35(0.09,1.41) | 2.38 | -1.50(-2.82) | 0.35(0.09) |
| Reproductive system and breast disorders | 2 | 0.12(0.03,0.47) | 0.12(0.03,0.48) | 13.08 | -3.06(-4.23) | 0.12(0.03) |
| Blood and lymphatic system disorders | 2 | 0.06(0.02,0.25) | 0.06(0.02,0.25) | 28.19 | -3.98(-5.11) | 0.06(0.02) |
| Endocrine disorders | 1 | 0.21(0.03,1.49) | 0.21(0.03,1.49) | 2.97 | -2.25(-3.57) | 0.21(0.03) |

Note: Ranked by case reports.

**Supplementary Table S6** Signal strength of adverse drug reactions at the Preferred Term (PT) level ranked by ROR

| **Preferred Term (PT)** | **Case reports** | **ROR (95% CI)** | **PRR (95% CI)** | **Chi Square** | **IC (IC025)** | **EBGM (EBGM05)** | **Corrected P value** |
| --- | --- | --- | --- | --- | --- | --- | --- |
| Vitamin A decreased | 6 | 4043.43  (1771.70,9228.05) | 4029.62  (1770.35,9172.09) | 22797.9 | 11.89  (1.68) | 3801.59  (1665.73) | 0 |
| Heart transplant | 13 | 1113.41  (642.34,1929.94) | 1105.17  (640.14,1908.01) | 14109.1 | 10.09  (3.01) | 1087.29  (627.27) | 0 |
| Cardiac amyloidosis | 6 | 425.17  (190.27,950.09) | 423.72  (190.14,944.26) | 2514.52 | 8.72  (1.69) | 421.07  (188.44) | 0 |
| Night blindness | 6 | 196.76  (88.17,439.08) | 196.09  (88.11,436.39) | 1161.19 | 7.61  (1.67) | 195.52  (87.62) | 0 |
| Cardiac ablation | 4 | 142.46  (53.35,380.40) | 142.14  (53.35,378.69) | 559.41 | 7.15  (0.99) | 141.84  (53.12) | 0 |
| Neck surgery | 3 | 92.66  (29.83,287.82) | 92.51  (29.84,286.78) | 271.19 | 6.53  (0.51) | 92.38  (29.74) | 0 |
| Cardiac pacemaker insertion | 8 | 85.10  (42.47,170.52) | 84.72  (42.42,169.21) | 661.04 | 6.40  (2.07) | 84.61  (42.23) | 0 |
| Renal transplant | 4 | 79.35  (29.73,211.79) | 79.18  (29.73,210.85) | 308.40 | 6.31  (0.96) | 79.08  (29.63) | 0 |
| Shoulder operation | 4 | 71.84  (26.92,191.72) | 71.68  (26.92,190.86) | 278.47 | 6.16  (0.95) | 71.60  (26.83) | 0 |
| Therapy interrupted | 50 | 64.97  (49.04,86.08) | 63.15  (48.04,83.00) | 3056.64 | 5.98  (4.42) | 63.09  (47.62) | 0 |
| Therapeutic response shortened | 18 | 52.55  (33.02,83.62) | 52.02  (32.84,82.38) | 900.14 | 5.70  (3.15) | 51.98  (32.66) | 0 |
| Fine motor skill dysfunction | 3 | 52.02  (16.75,161.51) | 51.93  (16.76,160.92) | 149.73 | 5.70  (0.47) | 51.89  (16.71) | 0 |
| Drug effect less than expected | 6 | 39.21  (17.59,87.42) | 39.08  (17.58,86.89) | 222.53 | 5.29  (1.51) | 39.06  (17.52) | 0 |
| Intentional dose omission | 11 | 36.12  (19.96,65.36) | 35.90  (19.92,64.72) | 373.08 | 5.17  (2.36) | 35.88  (19.83) | 0 |
| Hospitalisation | 63 | 36.01  (28.00,46.31) | 34.75  (27.27,44.29) | 2066.23 | 5.12  (4.14) | 34.73  (27.01) | 0 |
| Left ventricular failure | 3 | 35.57  (11.46,110.44) | 35.52  (11.46,110.05) | 100.58 | 5.15  (0.44) | 35.50  (11.43) | 0 |
| Knee arthroplasty | 9 | 34.84  (18.10,67.09) | 34.67  (18.07,66.53) | 294.18 | 5.11  (2.07) | 34.65  (18.00) | 0 |
| Knee operation | 4 | 32.24  (12.08,86.02) | 32.17  (12.08,85.64) | 120.75 | 5.01  (0.86) | 32.15  (12.05) | 0 |
| Spinal operation | 5 | 30.93  (12.86,74.43) | 30.85  (12.85,74.03) | 144.34 | 4.95  (1.19) | 30.83  (12.81) | 0 |
| Cardiac operation | 3 | 25.83  (8.32,80.18) | 25.78  (8.32,79.89) | 71.45 | 4.69  (0.40) | 25.78  (8.30) | 0 |
| Hypervolaemia | 6 | 20.23  (9.07,45.09) | 20.16  (9.07,44.82) | 109.25 | 4.33  (1.34) | 20.16  (9.04) | 0 |
| Carpal tunnel syndrome | 4 | 17.63  (6.61,47.03) | 17.59  (6.61,46.83) | 62.59 | 4.14  (0.73) | 17.59  (6.59) | 0 |
| Vitreous floaters | 3 | 16.22  (5.23,50.35) | 16.19  (5.23,50.17) | 42.76 | 4.02  (0.31) | 16.19  (5.22) | 0 |
| Hip arthroplasty | 3 | 15.67  (5.05,48.65) | 15.65  (5.05,48.48) | 41.13 | 3.97  (0.30) | 15.65  (5.04) | 0 |
| Dialysis | 3 | 13.43  (4.33,41.68) | 13.41  (4.33,41.53) | 34.44 | 3.74  (0.26) | 13.40  (4.32) | 0 |
| Lower limb fracture | 3 | 11.74  (3.78,36.45) | 11.72  (3.78,36.32) | 29.43 | 3.55  (0.23) | 11.72  (3.78) | 0 |
| Brain fog | 3 | 11.04  (3.56,34.26) | 11.02  (3.56,34.14) | 27.33 | 3.46  (0.21) | 11.02  (3.55) | 0 |
| Ill-defined disorder | 10 | 10.23  (5.49,19.04) | 10.17  (5.48,18.88) | 82.76 | 3.35  (1.60) | 10.17  (5.46) | 0 |
| Hip fracture | 4 | 9.49  (3.56,25.32) | 9.47  (3.56,25.21) | 30.32 | 3.24  (0.52) | 9.47  (3.55) | 0 |
| Surgery | 8 | 9.39  (4.69,18.81) | 9.35  (4.68,18.67) | 59.70 | 3.23  (1.31) | 9.35  (4.67) | 0 |
| Glaucoma | 3 | 9.39  (3.02,29.14) | 9.37  (3.03,29.04) | 22.44 | 3.23  (0.15) | 9.37  (3.02) | 4.28E-15 |
| Disability | 4 | 9.21  (3.45,24.56) | 9.19  (3.45,24.46) | 29.19 | 3.20  (0.51) | 9.19  (3.44) | 1.79E-14 |
| Neuropathy peripheral | 22 | 9.11  (5.98,13.87) | 9.01  (5.95,13.64) | 156.76 | 3.17  (2.13) | 9.00  (5.91) | 1.47E-13 |
| Cardiac failure | 12 | 8.76  (4.96,15.46) | 8.71  (4.95,15.30) | 81.91 | 3.12  (1.65) | 8.71  (4.93) | 9.46E-11 |
| Ventricular tachycardia | 3 | 8.40  (2.71,26.08) | 8.39  (2.71,25.99) | 19.53 | 3.07  (0.11) | 8.39  (2.70) | 1.08E-10 |
| Cardiac disorder | 12 | 7.61  (4.32,13.44) | 7.57  (4.31,13.30) | 68.48 | 2.92  (1.53) | 7.57  (4.29) | 2.05E-10 |
| Head injury | 4 | 7.44  (2.79,19.85) | 7.43  (2.79,19.77) | 22.25 | 2.89  (0.41) | 7.43  (2.78) | 6.19E-09 |
| Mental impairment | 4 | 7.44  (2.79,19.84) | 7.42  (2.79,19.76) | 22.24 | 2.89  (0.41) | 7.42  (2.78) | 3.33E-08 |
| Death | 97 | 7.19  (5.86,8.83) | 6.85  (5.65,8.31) | 488.70 | 2.78  (2.39) | 6.85  (5.58) | 4.89E-08 |
| Fluid retention | 6 | 7.06  (3.17,15.74) | 7.04  (3.17,15.65) | 31.11 | 2.82  (0.82) | 7.04  (3.16) | 7.55E-08 |
| Balance disorder | 12 | 6.50  (3.69,11.47) | 6.47  (3.68,11.36) | 55.50 | 2.69  (1.38) | 6.47  (3.66) | 8.32E-08 |
| Unevaluable event | 15 | 6.19  (3.73,10.30) | 6.15  (3.72,10.18) | 64.78 | 2.62  (1.49) | 6.15  (3.70) | 1.42E-07 |
| Blindness | 4 | 5.99  (2.25,15.98) | 5.98  (2.25,15.92) | 16.60 | 2.58  (0.29) | 5.98  (2.24) | 2.07E-07 |
| Disease progression | 11 | 5.71  (3.16,10.33) | 5.68  (3.15,10.24) | 42.45 | 2.51  (1.19) | 5.68  (3.14) | 2.17E-07 |
| Fall | 28 | 5.53  (3.81,8.04) | 5.46  (3.78,7.88) | 102.28 | 2.45  (1.70) | 5.46  (3.76) | 2.91E-07 |
| Gait disturbance | 21 | 5.35  (3.48,8.23) | 5.30  (3.47,8.11) | 73.44 | 2.41  (1.53) | 5.30  (3.45) | 2.45E-06 |
| Cardiac failure congestive | 7 | 4.96  (2.36,10.42) | 4.94  (2.36,10.36) | 22.04 | 2.31  (0.70) | 4.94  (2.35) | 2.65E-06 |
| Illness | 9 | 4.94  (2.56,9.51) | 4.92  (2.56,9.43) | 28.11 | 2.30  (0.91) | 4.92  (2.55) | 2.61E-06 |
| Atrial fibrillation | 9 | 4.83  (2.51,9.30) | 4.81  (2.51,9.23) | 27.20 | 2.27  (0.88) | 4.81  (2.50) | 2.83E-06 |
| Mobility decreased | 7 | 4.66  (2.22,9.78) | 4.64  (2.22,9.72) | 20.02 | 2.21  (0.65) | 4.64  (2.21) | 7.99E-06 |
| Product dose omission issue | 30 | 4.36  (3.04,6.25) | 4.30  (3.02,6.13) | 76.32 | 2.10  (1.44) | 4.30  (3.00) | 2.72E-05 |
| Limb discomfort | 7 | 4.30  (2.05,9.03) | 4.28  (2.05,8.97) | 17.64 | 2.10  (0.58) | 4.28  (2.04) | 4.62E-05 |
| Loss of consciousness | 12 | 3.98  (2.26,7.02) | 3.96  (2.25,6.96) | 26.59 | 1.99  (0.89) | 3.96  (2.24) | 0 |

Note1: Ranked by ROR

Note2: Signals are detected when all the following criteria are meta ≥ 3, PRR ≥2 and Chi-Square ≥ 4, lower limit of 95% CI of ROR > 1, IC025 > 0, EBGM05 > 2.

**Supplementary Table S7** Signal strength of adverse drug reactions at the Preferred Term (PT) level ranked by Reports

| **Preferred Term (PT)** | **Case reports** | **ROR (95% CI)** | **PRR (95% CI)** | **Chi Square** | **IC (IC025)** | **EBGM (EBGM05)** |
| --- | --- | --- | --- | --- | --- | --- |
| Death | 97 | 7.19  (5.86,8.83) | 6.85  (5.65,8.31) | 488.70 | 2.78  (2.39) | 6.85  (5.58) |
| Hospitalisation | 63 | 36.01  (28.00,46.31) | 34.75  (27.27,44.29) | 2066.23 | 5.12  (4.14) | 34.73  (27.01) |
| Therapy interrupted | 50 | 64.97  (49.04,86.08) | 63.15  (48.04,83.00) | 3056.64 | 5.98  (4.42) | 63.09  (47.62) |
| Product dose omission issue | 30 | 4.36  (3.04,6.25) | 4.30  (3.02,6.13) | 76.32 | 2.10  (1.44) | 4.30  (3.00) |
| Fall | 28 | 5.53  (3.81,8.04) | 5.46  (3.78,7.88) | 102.28 | 2.45  (1.70) | 5.46  (3.76) |
| Neuropathy peripheral | 22 | 9.11  (5.98,13.87) | 9.01  (5.95,13.64) | 156.76 | 3.17  (2.13) | 9.00  (5.91) |
| Gait disturbance | 21 | 5.35  (3.48,8.23) | 5.30  (3.47,8.11) | 73.44 | 2.41  (1.53) | 5.30  (3.45) |
| Therapeutic response shortened | 18 | 52.55  (33.02,83.62) | 52.02  (32.84,82.38) | 900.14 | 5.70  (3.15) | 51.98  (32.66) |
| Unevaluable event | 15 | 6.19  (3.73,10.30) | 6.15  (3.72,10.18) | 64.78 | 2.62  (1.49) | 6.15  (3.70) |
| Heart transplant | 13 | 1113.41  (642.34,1929.94) | 1105.17  (640.14,1908.01) | 14109.1 | 10.09  (3.01) | 1087.29  (627.27) |
| Loss of consciousness | 12 | 3.98  (2.26,7.02) | 3.96  (2.25,6.96) | 26.59 | 1.99  (0.89) | 3.96  (2.24) |
| Cardiac failure | 12 | 8.76  (4.96,15.46) | 8.71  (4.95,15.30) | 81.91 | 3.12  (1.65) | 8.71  (4.93) |
| Balance disorder | 12 | 6.50  (3.69,11.47) | 6.47  (3.68,11.36) | 55.50 | 2.69  (1.38) | 6.47  (3.66) |
| Cardiac disorder | 12 | 7.61  (4.32,13.44) | 7.57  (4.31,13.30) | 68.48 | 2.92  (1.53) | 7.57  (4.29) |
| Disease progression | 11 | 5.71  (3.16,10.33) | 5.68  (3.15,10.24) | 42.45 | 2.51  (1.19) | 5.68  (3.14) |
| Intentional dose omission | 11 | 36.12  (19.96,65.36) | 35.90  (19.92,64.72) | 373.08 | 5.17  (2.36) | 35.88  (19.83) |
| Ill-defined disorder | 10 | 10.23  (5.49,19.04) | 10.17  (5.48,18.88) | 82.76 | 3.35  (1.60) | 10.17  (5.46) |
| Illness | 9 | 4.94  (2.56,9.51) | 4.92  (2.56,9.43) | 28.11 | 2.30  (0.91) | 4.92  (2.55) |
| Atrial fibrillation | 9 | 4.83  (2.51,9.30) | 4.81  (2.51,9.23) | 27.20 | 2.27  (0.88) | 4.81  (2.50) |
| Knee arthroplasty | 9 | 34.84  (18.10,67.09) | 34.67  (18.07,66.53) | 294.18 | 5.11  (2.07) | 34.65  (18.00) |
| Cardiac pacemaker insertion | 8 | 85.10  (42.47,170.52) | 84.72  (42.42,169.21) | 661.04 | 6.40  (2.07) | 84.61  (42.23) |
| Surgery | 8 | 9.39  (4.69,18.81) | 9.35  (4.68,18.67) | 59.70 | 3.23  (1.31) | 9.35  (4.67) |
| Mobility decreased | 7 | 4.66  (2.22,9.78) | 4.64  (2.22,9.72) | 20.02 | 2.21  (0.65) | 4.64  (2.21) |
| Cardiac failure congestive | 7 | 4.96  (2.36,10.42) | 4.94  (2.36,10.36) | 22.04 | 2.31  (0.70) | 4.94  (2.35) |
| Limb discomfort | 7 | 4.30  (2.05,9.03) | 4.28  (2.05,8.97) | 17.64 | 2.10  (0.58) | 4.28  (2.04) |
| Drug effect less than expected | 6 | 39.21  (17.59,87.42) | 39.08  (17.58,86.89) | 222.53 | 5.29  (1.51) | 39.06  (17.52) |
| Vitamin A decreased | 6 | 4043.43  (1771.70,9228.05) | 4029.62  (1770.35,9172.09) | 22797.9 | 11.89  (1.68) | 3801.59  (1665.73) |
| Night blindness | 6 | 196.76  (88.17,439.08) | 196.09  (88.11,436.39) | 1161.19 | 7.61  (1.67) | 195.52  (87.62) |
| Fluid retention | 6 | 7.06  (3.17,15.74) | 7.04  (3.17,15.65) | 31.11 | 2.82  (0.82) | 7.04  (3.16) |
| Hypervolaemia | 6 | 20.23  (9.07,45.09) | 20.16  (9.07,44.82) | 109.25 | 4.33  (1.34) | 20.16  (9.04) |
| Cardiac amyloidosis | 6 | 425.17  (190.27,950.09) | 423.72  (190.14,944.26) | 2514.52 | 8.72  (1.69) | 421.07  (188.44) |
| Spinal operation | 5 | 30.93  (12.86,74.43) | 30.85  (12.85,74.03) | 144.34 | 4.95  (1.19) | 30.83  (12.81) |
| Shoulder operation | 4 | 71.84  (26.92,191.72) | 71.68  (26.92,190.86) | 278.47 | 6.16  (0.95) | 71.60  (26.83) |
| Cardiac ablation | 4 | 142.46  (53.35,380.40) | 142.14  (53.35,378.69) | 559.41 | 7.15  (0.99) | 141.84  (53.12) |
| Renal transplant | 4 | 79.35  (29.73,211.79) | 79.18  (29.73,210.85) | 308.40 | 6.31  (0.96) | 79.08  (29.63) |
| Disability | 4 | 9.21  (3.45,24.56) | 9.19  (3.45,24.46) | 29.19 | 3.20  (0.51) | 9.19  (3.44) |
| Knee operation | 4 | 32.24  (12.08,86.02) | 32.17  (12.08,85.64) | 120.75 | 5.01  (0.86) | 32.15  (12.05) |
| Carpal tunnel syndrome | 4 | 17.63  (6.61,47.03) | 17.59  (6.61,46.83) | 62.59 | 4.14  (0.73) | 17.59  (6.59) |
| Blindness | 4 | 5.99  (2.25,15.98) | 5.98  (2.25,15.92) | 16.60 | 2.58  (0.29) | 5.98  (2.24) |
| Hip fracture | 4 | 9.49  (3.56,25.32) | 9.47  (3.56,25.21) | 30.32 | 3.24  (0.52) | 9.47  (3.55) |
| Head injury | 4 | 7.44  (2.79,19.85) | 7.43  (2.79,19.77) | 22.25 | 2.89  (0.41) | 7.43  (2.78) |
| Mental impairment | 4 | 7.44  (2.79,19.84) | 7.42  (2.79,19.76) | 22.24 | 2.89  (0.41) | 7.42  (2.78) |
| Vitreous floaters | 3 | 16.22  (5.23,50.35) | 16.19  (5.23,50.17) | 42.76 | 4.02  (0.31) | 16.19  (5.22) |
| Ventricular tachycardia | 3 | 8.40  (2.71,26.08) | 8.39  (2.71,25.99) | 19.53 | 3.07  (0.11) | 8.39  (2.70) |
| Glaucoma | 3 | 9.39  (3.02,29.14) | 9.37  (3.03,29.04) | 22.44 | 3.23  (0.15) | 9.37  (3.02) |
| Left ventricular failure | 3 | 35.57  (11.46,110.44) | 35.52  (11.46,110.05) | 100.58 | 5.15  (0.44) | 35.50  (11.43) |
| Hip arthroplasty | 3 | 15.67  (5.05,48.65) | 15.65  (5.05,48.48) | 41.13 | 3.97  (0.30) | 15.65  (5.04) |
| Dialysis | 3 | 13.43  (4.33,41.68) | 13.41  (4.33,41.53) | 34.44 | 3.74  (0.26) | 13.40  (4.32) |
| Cardiac operation | 3 | 25.83  (8.32,80.18) | 25.78  (8.32,79.89) | 71.45 | 4.69  (0.40) | 25.78  (8.30) |
| Fine motor skill dysfunction | 3 | 52.02  (16.75,161.51) | 51.93  (16.76,160.92) | 149.73 | 5.70  (0.47) | 51.89  (16.71) |
| Neck surgery | 3 | 92.66  (29.83,287.82) | 92.51  (29.84,286.78) | 271.19 | 6.53  (0.51) | 92.38  (29.74) |
| Lower limb fracture | 3 | 11.74  (3.78,36.45) | 11.72  (3.78,36.32) | 29.43 | 3.55  (0.23) | 11.72  (3.78) |
| Brain fog | 3 | 11.04  (3.56,34.26) | 11.02  (3.56,34.14) | 27.33 | 3.46  (0.21) | 11.02  (3.55) |

Note1: Ranked by Reports

Note2: Signals are detected when all the following criteria are meta ≥ 3, PRR ≥2 and Chi-Square ≥ 4, lower limit of 95% CI of ROR > 1, IC025 > 0, EBGM05 > 2.

**Supplementary Table S8** Signal strength of adverse drug reactions at the Preferred Term (PT) level ranked by ROR

| **System Organ Class (SOC)** | **Preferred Term (PT)** | **Case reports** | **ROR (95% CI)** | **PRR (95% CI)** | **Chi Square** | **IC (IC025)** | **EBGM (EBGM05)** | **Corrected P value** |
| --- | --- | --- | --- | --- | --- | --- | --- | --- |
| Investigations | Vitamin A decreased | 5 | 1710.10  (693.13,4219.18) | 1705.54  (692.86,4198.36) | 8044.50 | 10.65  (1.36) | 1610.84  (652.90) | 0 |
| Surgical and medical procedures | Heart transplant | 13 | 480.44  (277.21,832.68) | 477.12  (276.33,823.82) | 6076.67 | 8.87  (2.99) | 469.41  (270.84) | 0 |
| Cardiac disorders | Cardiac amyloidosis | 7 | 233.62  (110.89,492.19) | 232.75  (110.78,489.00) | 1602.45 | 7.85  (1.93) | 230.91  (109.60) | 0 |
| Eye disorders | Night blindness | 4 | 77.12  (28.88,205.97) | 76.96  (28.88,205.10) | 299.10 | 6.26  (0.95) | 76.76  (28.74) | 0 |
| Surgical and medical procedures | Cardiac ablation | 3 | 59.47  (19.14,184.77) | 59.37  (19.14,184.14) | 171.82 | 5.89  (0.48) | 59.25  (19.07) | 0 |
| Immune system disorders | Amyloidosis | 3 | 57.09  (18.38,177.37) | 57.00  (18.38,176.77) | 164.73 | 5.83  (0.48) | 56.89  (18.31) | 0 |
| Surgical and medical procedures | Cardiac pacemaker insertion | 10 | 53.86  (28.91,100.32) | 53.57  (28.86,99.46) | 515.02 | 5.74  (2.34) | 53.48  (28.71) | 0 |
| Surgical and medical procedures | Neck surgery | 3 | 47.66  (15.34,148.04) | 47.58  (15.35,147.54) | 136.59 | 5.57  (0.47) | 47.51  (15.29) | 0 |
| Surgical and medical procedures | Renal transplant | 4 | 37.88  (14.19,101.10) | 37.80  (14.19,100.68) | 143.14 | 5.24  (0.88) | 37.75  (14.15) | 0 |
| Nervous system disorders | Fine motor skill dysfunction | 3 | 36.57  (11.78,113.58) | 36.52  (11.78,113.20) | 103.51 | 5.19  (0.44) | 36.47  (11.74) | 0 |
| Surgical and medical procedures | Shoulder operation | 4 | 35.14  (13.17,93.79) | 35.07  (13.17,93.40) | 132.24 | 5.13  (0.87) | 35.03  (13.13) | 0 |
| Surgical and medical procedures | Therapy interrupted | 51 | 31.70  (23.99,41.87) | 30.86  (23.54,40.46) | 1473.36 | 4.95  (3.89) | 30.83  (23.34) | 0 |
| Cardiac disorders | Cardiac failure chronic | 4 | 31.58  (11.83,84.28) | 31.52  (11.83,83.93) | 118.07 | 4.98  (0.86) | 31.48  (11.80) | 0 |
| Cardiac disorders | Left ventricular failure | 3 | 27.80  (8.95,86.31) | 27.75  (8.95,86.03) | 77.30 | 4.79  (0.41) | 27.73  (8.93) | 0 |
| Surgical and medical procedures | Eye operation | 3 | 24.56  (7.91,76.26) | 24.52  (7.91,76.00) | 67.64 | 4.61  (0.39) | 24.50  (7.89) | 0 |
| General disorders and administration site conditions | Therapeutic response shortened | 17 | 23.08  (14.31,37.22) | 22.88  (14.25,36.73) | 355.58 | 4.51  (2.68) | 22.86  (14.18) | 0 |
| General disorders and administration site conditions | Drug effect less than expected | 6 | 21.46  (9.63,47.85) | 21.40  (9.62,47.58) | 116.60 | 4.42  (1.36) | 21.38  (9.59) | 0 |
| Injury, poisoning and procedural complications | Intentional dose omission | 10 | 16.62  (8.93,30.94) | 16.54  (8.91,30.69) | 145.92 | 4.05  (1.90) | 16.53  (8.88) | 0 |
| Surgical and medical procedures | Knee arthroplasty | 9 | 15.61  (8.11,30.06) | 15.54  (8.10,29.83) | 122.45 | 3.96  (1.75) | 15.54  (8.07) | 0 |
| Surgical and medical procedures | Spinal operation | 5 | 15.38  (6.39,37.01) | 15.34  (6.39,36.83) | 67.01 | 3.94  (1.00) | 15.33  (6.37) | 0 |
| Surgical and medical procedures | Hospitalisation | 65 | 15.35  (11.98,19.66) | 14.85  (11.69,18.86) | 841.09 | 3.89  (3.25) | 14.84  (11.59) | 0 |
| Cardiac disorders | Cardiac failure acute | 3 | 15.32  (4.94,47.56) | 15.30  (4.94,47.40) | 40.07 | 3.93  (0.30) | 15.29  (4.93) | 0 |
| Surgical and medical procedures | Knee operation | 4 | 14.73  (5.52,39.30) | 14.70  (5.52,39.14) | 51.07 | 3.88  (0.68) | 14.70  (5.51) | 0 |
| Surgical and medical procedures | Cardiac operation | 3 | 12.04  (3.88,37.38) | 12.02  (3.88,37.26) | 30.31 | 3.59  (0.23) | 12.02  (3.87) | 0 |
| Injury, poisoning and procedural complications | Pelvic fracture | 3 | 11.61  (3.74,36.04) | 11.59  (3.74,35.93) | 29.03 | 3.53  (0.22) | 11.59  (3.73) | 0 |
| Metabolism and nutrition disorders | Hypervolaemia | 6 | 9.79  (4.39,21.83) | 9.77  (4.39,21.71) | 47.20 | 3.29  (1.02) | 9.76  (4.38) | 1.01E-12 |
| Vascular disorders | Peripheral coldness | 4 | 9.45  (3.54,25.21) | 9.43  (3.54,25.11) | 30.15 | 3.24  (0.52) | 9.43  (3.53) | 1.52E-12 |
| Nervous system disorders | Carpal tunnel syndrome | 4 | 9.39  (3.52,25.04) | 9.37  (3.52,24.94) | 29.90 | 3.23  (0.52) | 9.37  (3.51) | 9.5E-12 |
| General disorders and administration site conditions | Sudden death | 3 | 8.76  (2.82,27.18) | 8.74  (2.82,27.09) | 20.57 | 3.13  (0.13) | 8.74  (2.82) | 1.01E-11 |
| Cardiac disorders | Cardiac failure | 20 | 8.22  (5.29,12.77) | 8.14  (5.27,12.59) | 125.43 | 3.03  (1.97) | 8.14  (5.24) | 1.02E-11 |
| Nervous system disorders | Neuropathy peripheral | 22 | 7.88  (5.18,12.00) | 7.80  (5.15,11.82) | 130.55 | 2.96  (1.98) | 7.80  (5.12) | 3.63E-10 |
| Musculoskeletal and connective tissue disorders | Limb discomfort | 7 | 7.40  (3.52,15.55) | 7.38  (3.52,15.46) | 38.61 | 2.88  (1.01) | 7.38  (3.51) | 6.9E-10 |
| Surgical and medical procedures | Hip arthroplasty | 3 | 7.07  (2.28,21.93) | 7.06  (2.28,21.86) | 15.59 | 2.82  (0.04) | 7.05  (2.27) | 7.21E-10 |
| Eye disorders | Glaucoma | 4 | 6.83  (2.56,18.22) | 6.82  (2.56,18.15) | 19.87 | 2.77  (0.36) | 6.82  (2.56) | 5.42E-09 |
| Surgical and medical procedures | Dialysis | 3 | 6.76  (2.18,20.97) | 6.75  (2.18,20.90) | 14.69 | 2.75  (0.02) | 6.75  (2.17) | 3.29E-08 |
| Social circumstances | Disability | 4 | 6.75  (2.53,17.99) | 6.73  (2.53,17.92) | 19.53 | 2.75  (0.36) | 6.73  (2.52) | 4.7E-08 |
| Nervous system disorders | Neuralgia | 4 | 5.48  (2.05,14.61) | 5.47  (2.05,14.56) | 14.61 | 2.45  (0.24) | 5.47  (2.05) | 4.97E-08 |
| General disorders and administration site conditions | Unevaluable event | 13 | 5.47  (3.17,9.43) | 5.44  (3.16,9.35) | 47.12 | 2.44  (1.27) | 5.44  (3.15) | 5.52E-08 |
| Nervous system disorders | Mental impairment | 4 | 5.35  (2.00,14.26) | 5.34  (2.01,14.21) | 14.10 | 2.42  (0.22) | 5.34  (2.00) | 8.38E-08 |
| Surgical and medical procedures | Surgery | 8 | 4.98  (2.49,9.97) | 4.96  (2.48,9.91) | 25.31 | 2.31  (0.82) | 4.96  (2.48) | 5.6E-07 |
| Nervous system disorders | Hypoaesthesia | 22 | 4.78  (3.14,7.27) | 4.73  (3.12,7.17) | 64.92 | 2.24  (1.42) | 4.73  (3.11) | 5.75E-06 |
| Cardiac disorders | Cardiac disorder | 13 | 4.46  (2.58,7.69) | 4.43  (2.58,7.62) | 34.62 | 2.15  (1.06) | 4.43  (2.57) | 8.46E-06 |
| General disorders and administration site conditions | Ill-defined disorder | 8 | 4.38  (2.19,8.78) | 4.37  (2.19,8.72) | 20.79 | 2.13  (0.70) | 4.37  (2.18) | 8.89E-06 |
| General disorders and administration site conditions | Death | 105 | 4.23  (3.48,5.15) | 4.05  (3.36,4.88) | 244.75 | 2.02  (1.69) | 4.05  (3.33) | 1.04E-05 |
| General disorders and administration site conditions | Disease progression | 14 | 3.98  (2.36,6.74) | 3.96  (2.35,6.68) | 31.06 | 1.99  (0.98) | 3.96  (2.34) | 0.000135 |
| General disorders and administration site conditions | Gait disturbance | 23 | 3.83  (2.54,5.77) | 3.79  (2.53,5.69) | 47.40 | 1.92  (1.17) | 3.79  (2.51) | 0.000173 |
| Nervous system disorders | Balance disorder | 10 | 3.74  (2.01,6.97) | 3.73  (2.01,6.92) | 20.01 | 1.90  (0.71) | 3.73  (2.00) | 0 |
| Injury, poisoning and procedural complications | Fall | 33 | 3.30  (2.34,4.65) | 3.26  (2.32,4.57) | 51.94 | 1.70  (1.11) | 3.26  (2.31) | 0 |
| Musculoskeletal and connective tissue disorders | Pain in extremity | 28 | 3.08  (2.12,4.47) | 3.05  (2.11,4.40) | 38.76 | 1.61  (0.97) | 3.05  (2.10) | 0 |

Note1: Ranked by ROR.

Note2: Signals are detected when all the following criteria are meta ≥ 3, PRR ≥2 and Chi-Square ≥ 4, lower limit of 95% CI of ROR > 1, IC025 > 0, EBGM05 > 2.
